# Caregiver Depression, Change in the Severity of Neuropsychiatric Symptoms, and Subsequent Caregiver Abuse

**DOI:** 10.1093/geroni/igaa057.1127

**Published:** 2020-12-16

**Authors:** Boye Fang, Elsie Yan, Yaolin Pei, Gengzhen Chen

**Affiliations:** 1 Sun Yat-Sen University, Guangzhou, China; 2 The Hong Kong Polytechnic University, Hong Kong, China; 3 New York University, New York, New York, United States; 4 Shantou University Medical College, Shantou, China

## Abstract

Objectives: To examine whether caregiver (CG) depression predicts subsequent occurrence of elder abuse and whether change in the severity how care-recipient change in the severity of care recipient (CR) neuropsychiatric symptoms influence this association. Methods: Using two-year longitudinal data, we analyzed a consecutive sample of 800 Chinese family CGs and their CRs with dementia recruited from the geriatric and neurological departments of three Grade-A hospitals in People’s Republic of China (PRC). All the participatory dyads were assessed between September 2015 and February 2016 and followed for two years. Results: CG depression at baseline predicted the subsequent occurrence of physical and psychological abuse and CG neglect. However, change in the severity of CR neuropsychiatric symptoms altered these relationships. Specifically, while unchanged and increased CR neuropsychiatric symptoms heightened the positive effect of CG depression on subsequent abuse, decreased CR neuropsychiatric symptoms protected older adults from abuse by a depressed CG. Conclusion: This study showed the differential impact of CG depression on subsequent occurrence of elder abuse depending on the change in the severity of neuropsychiatric symptoms related to the CRs. The present findings provide important insights to the design of a systematic and integrative intervention protocol for elder abuse that simultaneously focuses on treating CG depression and CR neuropsychiatric symptoms.

